# Multiple myeloma with plasmacytoma of the clivus bone presenting with multiple cranial nerve III, IV, and VI palsy: A diagnostic dilemma

**DOI:** 10.1002/ccr3.6958

**Published:** 2023-02-13

**Authors:** Chiranjiwi Prasad Shah, Rajan Chamlagain, Sangam Shah, Subodh Paudel, Sanjit Kumar Sah, Bipin Koirala, Kamal Pandit, Sanjeeta Sitaula, Anjan Shrestha

**Affiliations:** ^1^ Maharajgunj Medical Campus, Institute of Medicine Tribhuvan University Maharajgunj Nepal; ^2^ Tribhuvan University Teaching Hospital Maharajgunj Nepal; ^3^ Department of Ophthalmology, Institute of Medicine Tribhuvan University Maharajgunj Nepal; ^4^ Department of Internal Medicine, Institute of Medicine Tribhuvan University Maharajgunj Nepal

**Keywords:** clivus bone, multiple myeloma, palsy, plasmacytoma

## Abstract

Central nervous system (CNS) manifestation with cranial nerve palsy in multiple myeloma (MM) is a rare manifestation. Plasmacytoma originates from the bones of the skull base in 3% patients with MM but rarely develops from the soft tissues of the nasal cavity and paranasal sinuses. Here, we present a case of 68‐year‐old male patient with multiple myeloma, clivus bone plasmacytoma, and cavernous sinus syndrome.

## Abstract 2

Clivus bone plasmacytoma with MM is a rare condition. Our patient presented with the pain in frontal and periorbital region, blurry vision and backache. Any patient presenting with these symptoms along with lytic bone lesions on imaging should be evaluated for MM.

## 1 INTRODUCTION

Multiple myeloma (MM) is characterized by chronic, progressive neoplastic proliferation of plasma cells that produces monoclonal immunoglobulin. Plasmacytomas are tumors of plasma cells that can arise in tissues (extramedullary plasmacytoma) or bones (osseous plasmacytoma).^1^ Central nervous system (CNS) manifestation with cranial nerve palsy in MM is a rare if it originates from the bones of the skull base. Likewise, plasmacytoma can uncommonly originate from the soft tissues of the nasal cavity and paranasal sinuses or it can be intracranial in about 3% patients.^2^, ^3^ The prognosis in patients with solitary bone plasmacytoma is poor as compared to solitary extramedullary plasmacytoma. Here, we present a case of 68 years, male patient, with cavernous sinus syndrome associated with multiple myeloma with solitary plasmacytoma of the clivus bone.

## 2 CASE PRESENTATION

A 68‐year‐old hypertensive male patient presented with gradual and progressive blurry vision and moderate periorbital pain for three months. He also complained of frontal headache which was dull, continuous, and non‐radiating, without any aggravating or relieving factors. He also complained of mild, intermittent backache aggravated by movement, and relieved by prolonged bedrest. He denied history of vomiting, tinnitus, or weight loss. He was not taking medications. He consumed 25 units of alcohol per week for the last 3 years but did not smoke cigarettes.

**FIGURE 1:**
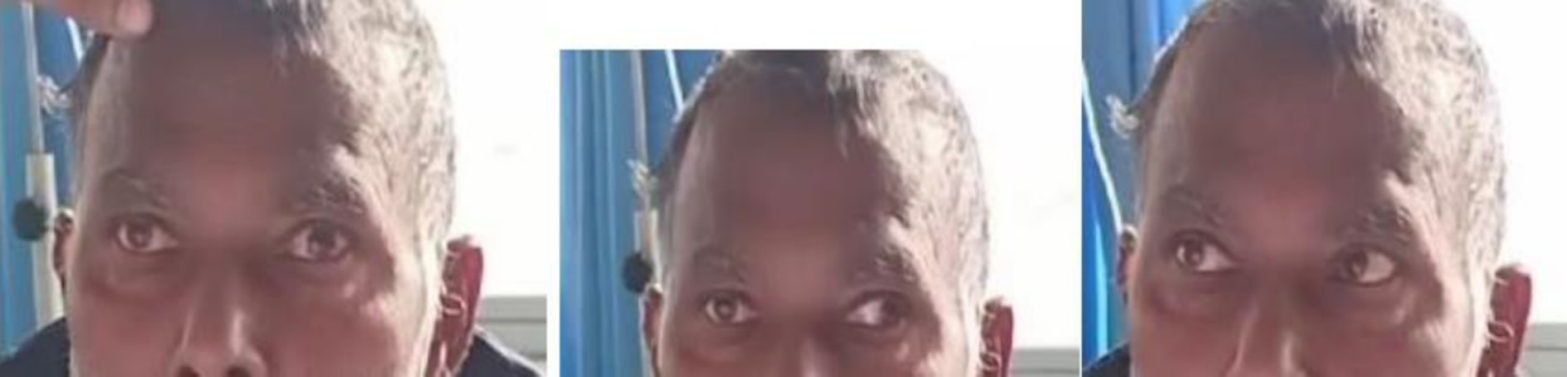
Figure showing multiple cranial nerve palsies of right eye

On ophthalmological evaluation, the patient had bilateral best‐corrected visual acuity of 6/24, restricted ocular motility in all cardinal gazes, and mild ptosis(2 mm) in the right eye. Findings included exotropia on the right eye (30 prism diopters), right mild hypertropia (2 prism diopter) with excyclotorsion (5 degrees) suggesting 3rd, 4th, and 6th cranial nerve (CN) palsy of the right eye (Figure 1). Pupillary examination, anterior segment, and corneal sensation were normal bilaterally. He had a bilateral grade II cortical cataractous lens. Fundoscopy revealed grossly normal findings. Color vision and contrast sensitivity tests were within the normal range. Hess's charting showed multiple nerve palsy in the right eye. Cranial nerve 5th, 7th, 8th, 9th, 10th, and 11th were intact. On the basis of history and examination, right‐sided complete external ophthalmoplegia with cavernous sinus lesion was suspected.

**FIGURE 2:**
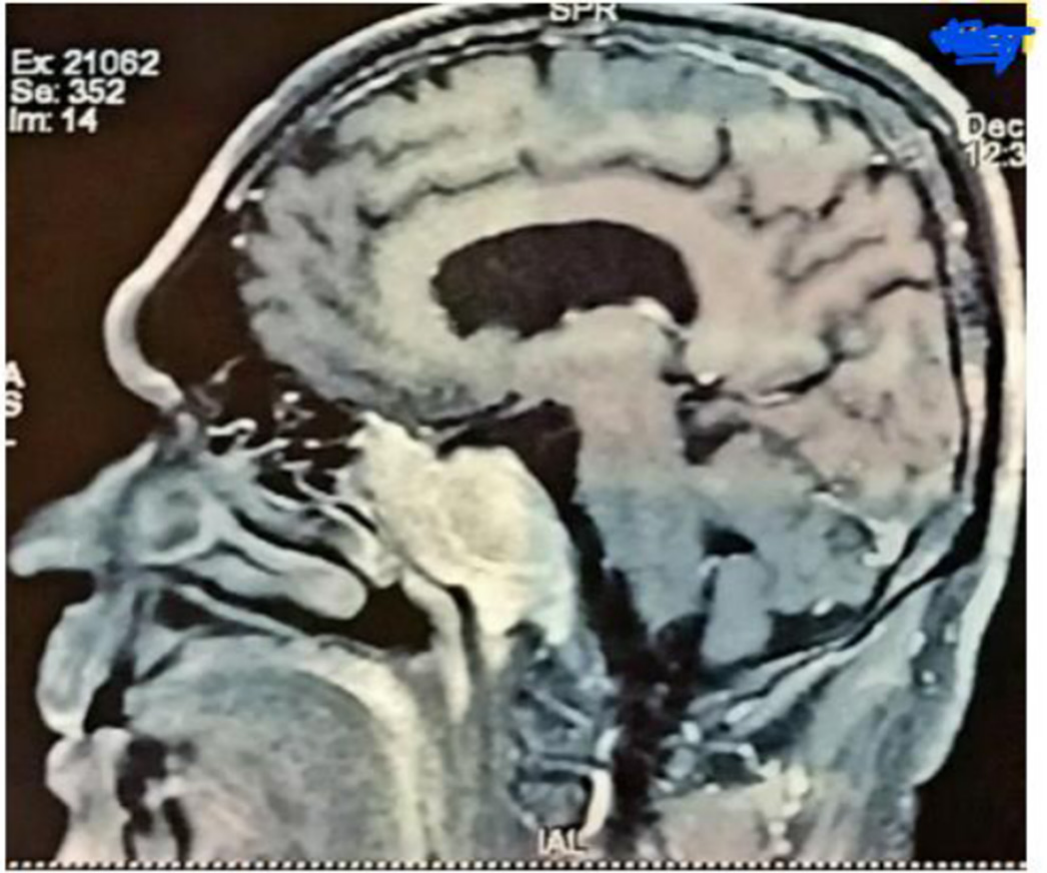
Brain magnetic resonance imaging (MRI) showing mass centered on clivus extending antero‐superiorly to the pituitary fossa, into bilateral sphenoid sinuses with their complete opacification and into bilateral ethmoid air cells and abutted bilateral optic nerves but had normal optic chiasma

Brain magnetic resonance imaging (MRI) with contrast showed 5.8 × 3.6 × 4.2 cm mass centered on clivus extending anterosuperior to the pituitary fossa, into bilateral sphenoid sinuses with their complete opacification, and into bilateral ethmoid air cells and abutted bilateral optic nerves but had normal optic chiasma (Figure 2). Antero‐inferiorly, the lesion had abutted the nasopharyngeal wall, laterally to cavernous sinuses, and posteriorly to the pre‐pontine cistern.

**FIGURE 3:**
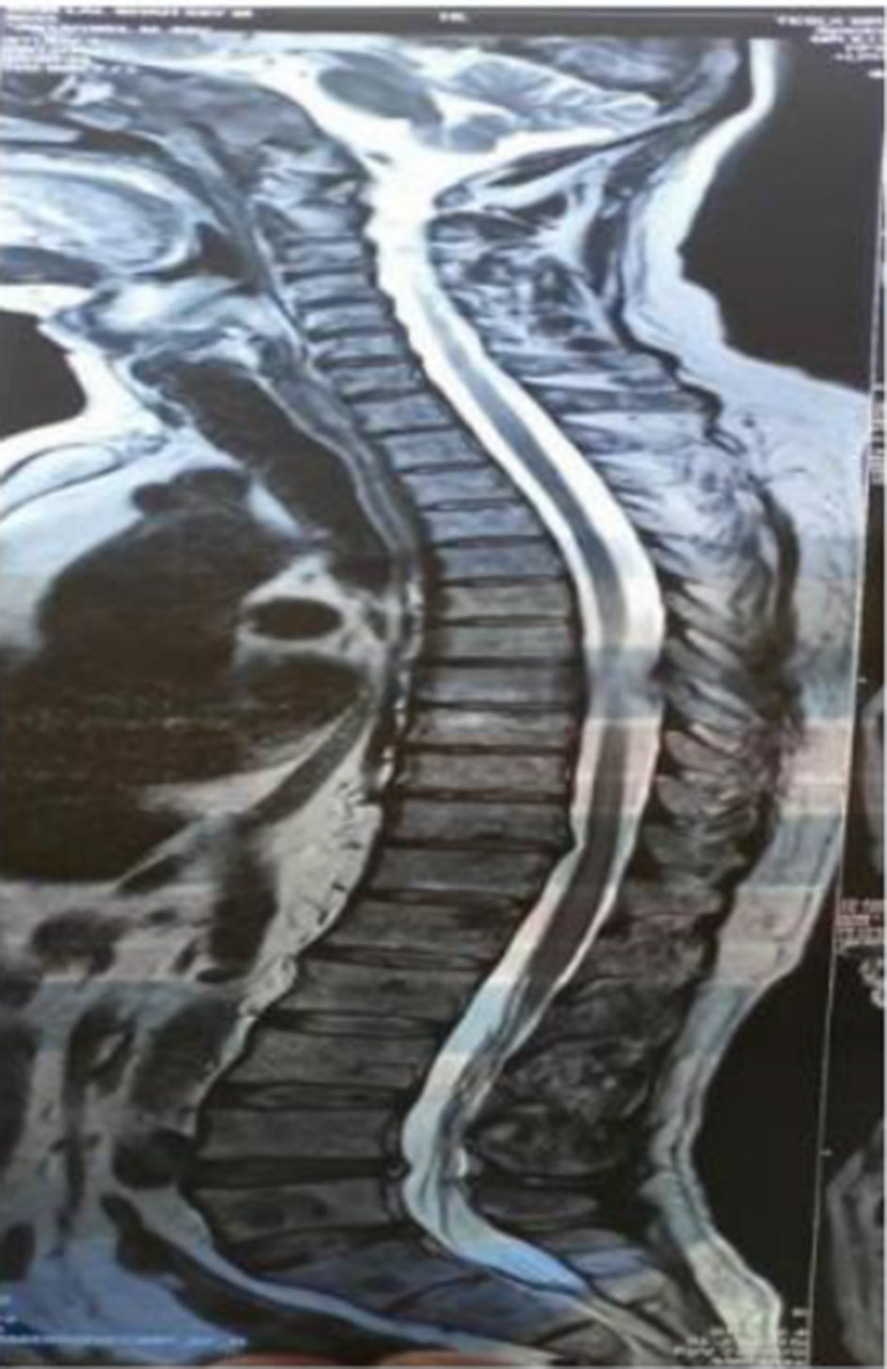
MRI of the whole spine revealed high signal intensity mass replacing the clivus

Contrast‐enhanced computer tomography (CECT) showed grossly normal findings in the chest and abdomen. Lytic lesion measuring 1.4 × 2.2 cm in the vertebral body of L5 with sclerotic rim and degenerative changes was found in the CT spine. MRI of the whole spine revealed high signal intensity mass replacing the clivus and variable‐sized areas of marrow replacement in multiple cervical, dorsal, and sacral vertebrae were suspicious for skeletal metastasis (Figure 3).

**FIGURE 4:**
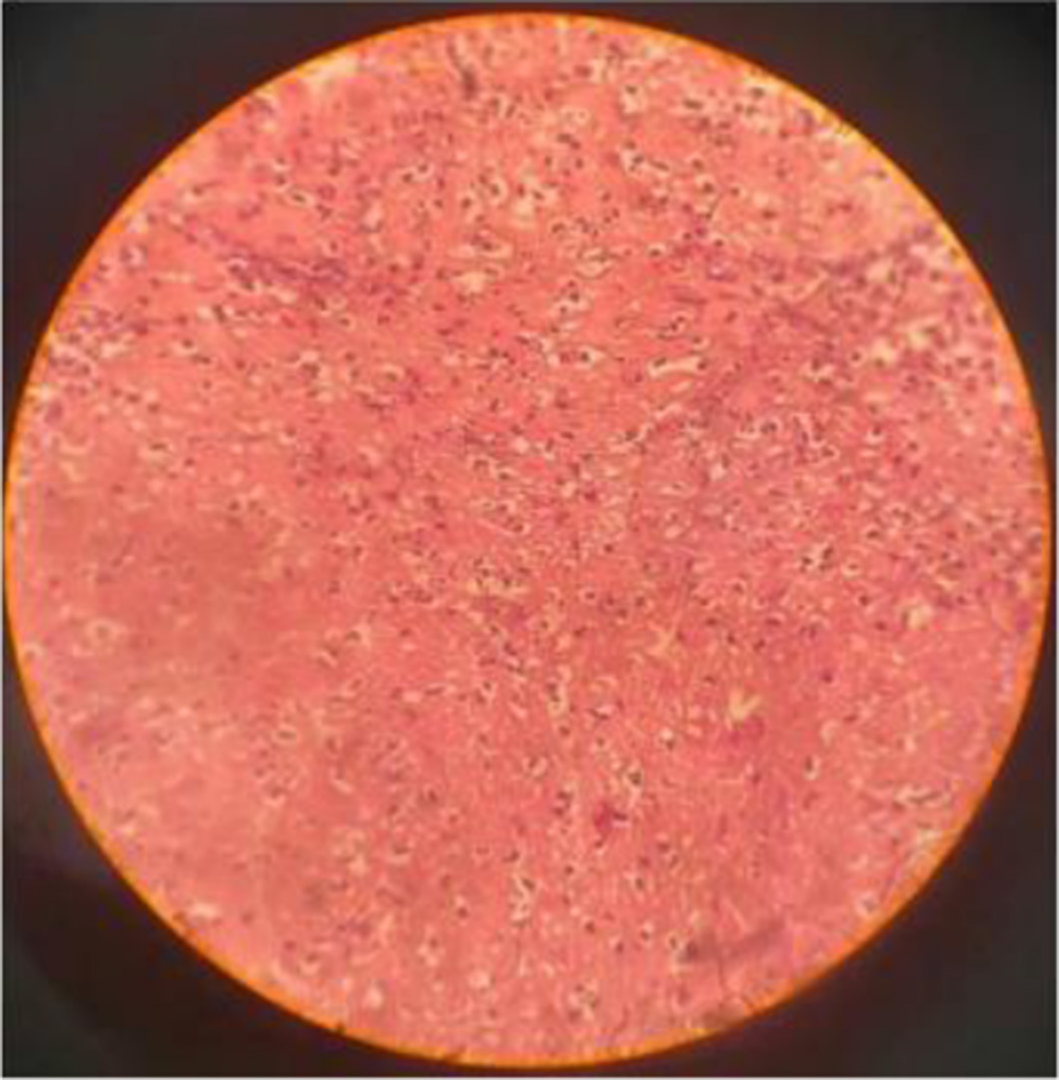
Biopsy showing predominant plasma cells

Furthermore, a bone marrow biopsy was done which demonstrated plasmacytosis with 58% plasma cells (Figure 4). Hematological investigations revealed hypercalcemia (calcium 11.2 mg/dL), increased urea and creatinine levels, anemia (hemoglobin 11.2 gm %) and liver function test was deranged. Total protein was increased to the level of 7.7 mg/dL. Likewise, serum electrophoresis revealed a monoclonal gamma peak. Finally, the diagnosis of multiple myeloma with clivus bone plasmacytoma was confirmed.

**FIGURE 5:**
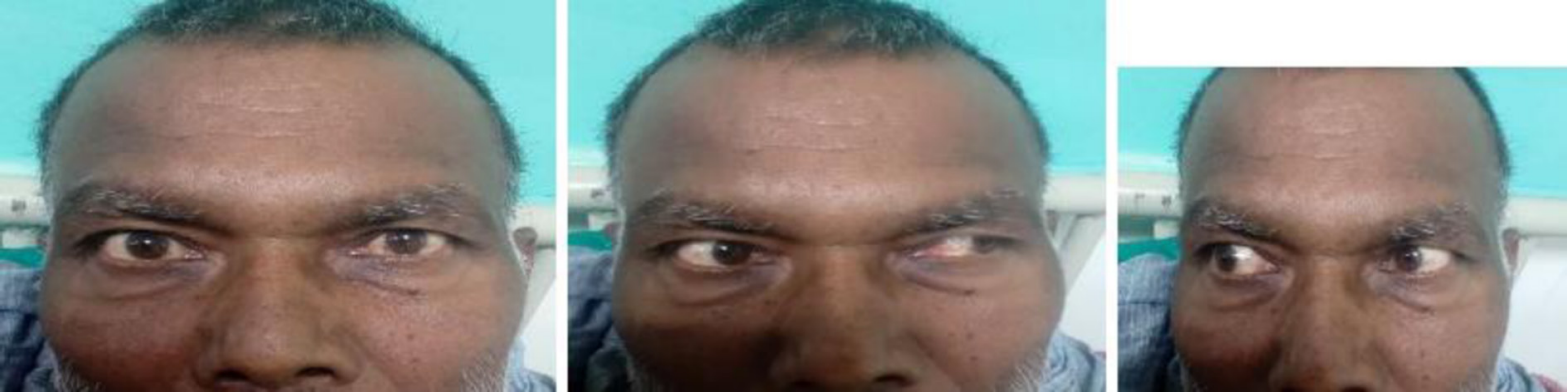
Figure showing improvement in the extraocular motility with no restriction in all gazes

The patient was admitted and treated with cyclophosphamide, bortezomib, and dexamethasone. The patient gradually improved over the hospital stay of three weeks. The extraocular motility was significantly improved with no restriction in any gazes (Figure 5). and bilateral visual acuity improved to 6/12.

## 3 DISCUSSION

Intracranial plasmacytomas are rare plasma cell tumors that originate from the skull, meninges, and brain. They only account for about 1% of all cerebral tumors. Only 3% of 273 patients with MM manifested with cerebral plasmacytoma, according to a study by Silverstein et al.^2^ To determine the origin and involvement of plasmacytomas, Clarke classified them into three groups: Group 1 includes plasmacytomas arising from the skull base with cranial nerve palsies; Group 2 includes plasmacytomas that involve the brain parenchyma with or without skull involvement and are referred to as “intracranial tumor syndromes”; and Group 3 includes intracranial tumor syndromes.^4^

Multiple myeloma (MM) is believed to be caused by the malignant transformation of post‐germinal center plasma cells, which produce monoclonal immunoglobulin, whereas plasmacytoma is plasma cell tumor.^5^ Intracranial plasmacytoma can arise from clivus bone, petrous part of the temporal bone and sino‐nasal or submucosa of the nasopharyngeal region. Plasmacytoma most commonly occurs in the sphenoid body and the apex of the petrous bones.^6^ Chordoma, chondrosarcoma, and meningioma are all possible diagnoses for a clival mass. Chondrosarcoma, osteosarcoma, and Ewing's tumor are the differential diagnoses for tumors emerging from the petrous apex and parasellar region. So, the proper investigation is required for the diagnosis of the disease. For visualizing the tumor, an MRI is superior to a CT scan, while a CT scan is ideal for determining the destruction of the affected bones.^7^ In a systematic review of 65 patients with sellar and clival plasmacytoma, 16% of patients were already diagnosed with MM, whereas 37% of patients were diagnosed with parasellar plasmacytoma with MM at the time of presentation.^8^

Patients may present with the symptoms of headaches, seizures, focal neurologic abnormalities, or cranial nerve palsy. There are various ophthalmic presentations of MM which includes proptosis, conjunctival involvement, corneal deposits, obstructed choriocapillaris, and hyperviscosity retinopathy. CNS manifestations include confusion, altered consciousness, gait disorder, and cranial nerve palsy. Cranial nerve palsy can lead to diplopia and visual disturbances. The most common type of unilateral right sixth cranial nerve palsy mentioned in the literature is isolated unilateral right sixth cranial nerve palsy.^9^ Very large tumors can also affect CN 9, 10, or 11.^10^

The molecular categorization of MM can provide clinically valuable information about prognosis and treatment stratification. Specific chromosomal high‐risk anomalies, such as t(4;14), t(14;16), t(14;20), del(17p), and dup(1q), have been linked to a poor prognosis previously. Nonetheless, both situations have a poor prognosis and should be treated aggressively.^11^, ^12^ We were unable to conduct the molecular categorization tests on our patients.

Cavernous sinus syndrome can be one of the common differential diagnoses if patients present with headaches, ophthalmoplegia, and visual disturbances. So, imaging modalities like CT/MRI can be helpful to distinguish it from other pathologies.

Multiple myeloma can be treated effectively with Bortezomib, Cyclophosphamide, and Dexamethasone (VCD chemotherapy) in patients who are not eligible for bone marrow transplants. While on active therapy, elderly patients required dose changes and particular considerations to balance the efficacy and toxicity of various drug combinations.^13^

## 4 CONCLUSION

Plasmacytoma is rare and clivus bone plasmacytoma with MM is an even more rare entity. Our patient presented with the symptoms of orbital and frontal region pain, blurry vision, and backache. Any patient presenting with these symptoms and an associated lytic bone lesion on imaging should be evaluated for MM. The diagnostic dilemma can occur as the patient can present with features of ophthalmoplegia. So, the multidisciplinary team approach can be crucial in the treatment of these patients.

## AUTHORS' CONTRIBUTION

CPS, RC, and SS wrote the original manuscript, and reviewed, and edited the manuscript. SKS, SP, SS, AS, BK, and KP reviewed the manuscript and reviewed it for publication. SS, AS, KP, BK, SKS, RC, SP, SS, CPS, BK, and KP were in charge of the case.

## CONFLICT OF INTEREST

Authors have no conflict of interest to declare.

## CONSENT

Written informed consent was obtained from the patient for the publication.

## Data Availability

All the required information is in manuscript itself.
